# Exploring the Potential of Novel Animal-Origin Probiotics as Key Players in One Health: Opportunities and Challenges

**DOI:** 10.3390/ijms26115143

**Published:** 2025-05-27

**Authors:** Zofia Gorzelanna, Aleksandra Mamrot, Daria Będkowska, Joanna Bubak, Marta Miszczak

**Affiliations:** 1 EZA Student Science Club, Department of Epizootiology and Clinic of Birds and Exotic Animals, Division of Infectious Diseases and Veterinary Administration, The Faculty of Veterinary Medicine, Wrocław University of Environmental and Life Sciences, Grunwaldzki Sq. 45., 50-366 Wrocław, Poland; 118512@student.upwr.edu.pl (Z.G.); 120374@student.upwr.edu.pl (A.M.); 118245@student.upwr.edu.pl (D.B.); 2 Department of Pathology, Division of Pathomorphology and Veterinary Forensics, The Faculty of Veterinary Medicine, Wrocław University of Environmental and Life Sciences, 31 Norwida St., 50-375 Wrocław, Poland; joanna.bubak@upwr.edu.pl; 3 Department of Epizootiology and Clinic of Birds and Exotic Animals, Division of Infectious Diseases and Veterinary Administration, The Faculty of Veterinary Medicine, Wrocław University of Environmental and Life Sciences, Grunwaldzki Sq. 45., 50-366 Wrocław, Poland

**Keywords:** probiotics, beneficial bacteria, human, animal, microbiota-host interaction, animal-derived strains, therapeutic potential, livestock, companion animals

## Abstract

Probiotics play a critical role in promoting the health of both humans and animals, with growing interest in the potential of animal-derived strains. Safety and efficacy assessments are crucial, with rigorous testing required to ensure the absence of harmful effects. The health benefits of animal-derived probiotic strains include improved digestion, balanced microbiota, behavioral impact, reduced inflammation, and minimized risk of infections. Probiotics of animal origin show promise as complementary or alternative options to antibiotics, with potential applications in both veterinary and human medicine. While promising, the usage of animal-derived probiotics requires careful evaluation of safety and regulatory aspects. This research underscores their potential for promoting health across species and contributing to future therapeutic approaches.

## 1. Introduction

Probiotics are living microorganisms that, when administered in adequate amounts, may confer a health benefit on the host [1]. Probiotic bacteria have been demonstrated to exert only a marginal effect on microbial diversity and typically fail to establish persistent colonization in the gut due to competitive exclusion by the resident microbiota [2]. Probiotic-derived components, such as bacteriocins, amines, and hydrogen peroxide, interact with specific targets within these pathways, thereby regulating apoptosis, cell proliferation, inflammation, and differentiation [3]. A prebiotic is a non-digestible compound that, when metabolized by gut microorganisms, influences the composition and activity of the gut microbiota, thereby providing a beneficial physiological effect on the host [4]. Prebiotics, by serving as substrates for beneficial microbes, promote a favorable gut environment, leading to the production of short-chain fatty acids (SCFAs) such as butyrate, propionate, and acetate [5]. Synbiotics are formulations that combine probiotics and prebiotics in a synergistic manner. The prebiotic component supports the survival and activity of the probiotic strains, enhancing their efficacy in modulating the gut microbiota and conferring health benefits [6]. The term “postbiotic” denotes the formulation of inactivated bacteria and/or their components in a way that supports host health [7]. Postbiotics comprise bioactive compounds produced during the fermentation of probiotic bacteria [8].

**Figure 1:**
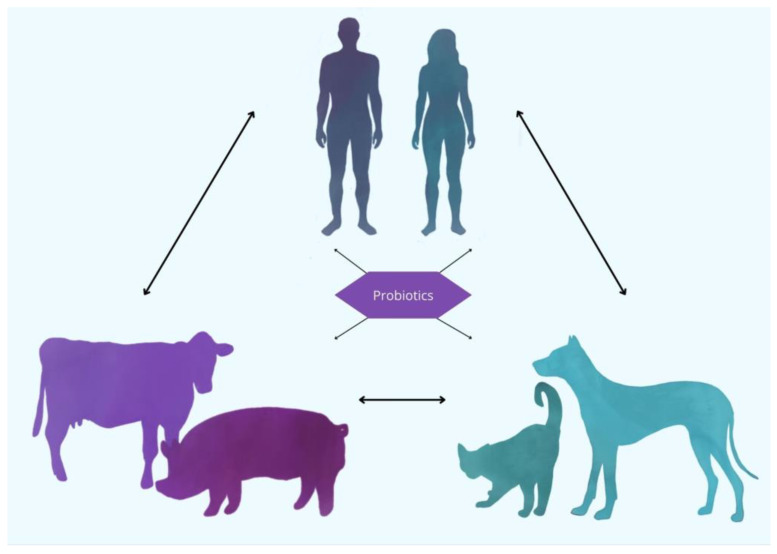
Probiotics as a unifying factor in One Health: connecting humans, pets, and livestock. The use of probiotics can serve as a bridge, promoting a shared microbial ecosystem across different domains. By modulating the gut microbiota in humans, pets, and livestock, probiotics contribute to enhanced health outcomes and disease prevention. This integrated perspective underscores the importance of considering all species in the context of health interventions, where probiotics can play a vital role in maintaining a balanced microbiome, reducing the transmission of infectious diseases, and improving overall well-being across species.

Probiotic bacterial strains are specific microorganisms identified and selected for their beneficial effects on host health. Historically, these strains were isolated from traditional fermented foods and the intestines of healthy individuals. The utilization of fermented foods for health benefits has a long history, spanning thousands of years. Fermented dairy products, particularly yogurt, were widely consumed by nomadic societies due to their portability, extended shelf-life, and the health benefits attributed to their probiotic content [9]. This historical precedent underscores the enduring connection between probiotics and human health, with contemporary research now substantiating and refining these early observations. As a result, probiotics have transitioned from traditional remedies to scientifically validated agents for promoting gastrointestinal health and overall well-being [10]. Advancements in microbiological techniques have significantly enhanced the ability to isolate probiotic strains from a diverse range of sources, including human breast milk and the gastrointestinal tracts of animals. These modern techniques, such as genomic sequencing, metagenomics, and high-throughput screening, allow for the precise identification, characterization, and validation of microbial strains, ensuring their potential efficacy and safety for therapeutic applications [11]. The growing body of research surrounding animal-derived probiotics highlights their potential as valuable therapeutic agents, not only for human health but also in the context of veterinary medicine, animal husbandry, and the One Health concept [12] (Figure 1). Probiotic strains derived from animals have garnered increasing interest due to their potential health benefits. The gastrointestinal tracts of various animal species, including pigs, poultry, and even bees, have been identified as sources of beneficial microorganisms with probiotic potential. Animal-derived probiotics, defined as microbial strains isolated from the gastrointestinal tracts or mucosal surfaces of animals, have garnered increasing attention due to their potential to confer host-specific health benefits. Certain animal-derived *Bifidobacterium* species have demonstrated beneficial effects on gut microbiota composition, gut barrier integrity, and immune system modulation [13]. In addition, studies have shown that probiotics derived from the gastrointestinal microbiota of bees exhibit antimicrobial properties and can enhance the health and immune responses of the host [14]. As scientific understanding of these probiotics continues to expand, the therapeutic applications of probiotics derived from animal sources may play a more significant role in promoting gut health, immune function, and overall well-being. Despite these promising attributes, research on animal-derived probiotics remains relatively limited. In particular, more studies are needed to elucidate their strain-specific mechanisms of action, host interactions, and long-term effects, especially in the context of cross-species application. Additionally, the lack of standardized evaluation criteria and insufficient genomic characterization of many strains contribute to significant knowledge gaps in this field. Addressing these issues is crucial for advancing their safe and effective use in both veterinary and human medicine.

## 2. Probiotics’ Role in Promoting Health in Humans and Animals

**Figure 2:**
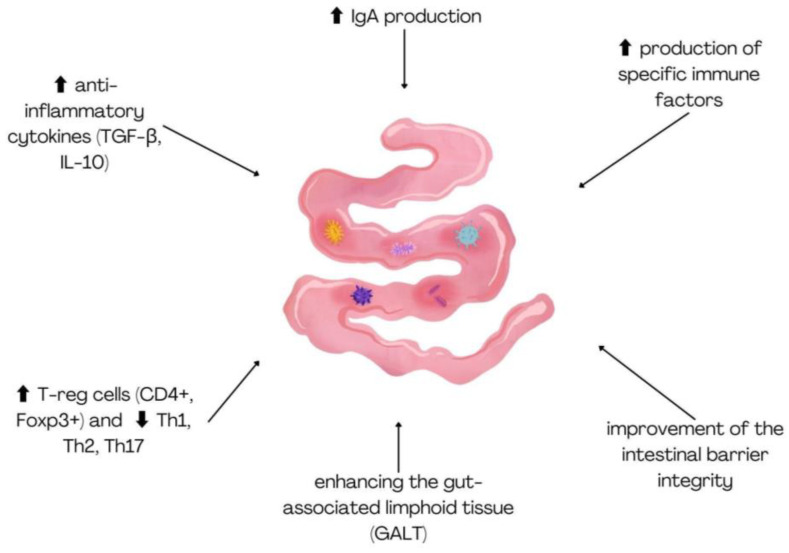
Immunomodulatory effects of animal-derived probiotic strains on gut immunity.

Probiotics offer a wide range of health benefits for both humans and animals, from improving digestion to supporting immune function and mental health. While antibiotics are used sub-therapeutically in animal feed to promote growth, excessive or improper use can lead to the development of antibiotic-resistant bacteria [15]. This overuse of antibiotics has become a major public health concern due to its potential to cause both human and animal diseases. In addition to the risks posed by antibiotic overuse, foodborne pathogenic bacteria are responsible for major zoonotic diseases such as salmonellosis, campylobacteriosis, and infections caused by pathogenic *Escherichia coli* in humans [16]. To address these challenges in livestock and aquaculture production, probiotics have emerged as an effective alternative, promoting animal growth and health without the negative side effects associated with antibiotics. Today, probiotic feed supplements are commonly provided to poultry, ruminants, and fish. These probiotics are mostly Gram-positive bacteria, although Gram-negative bacteria, yeast, and fungi are also used [17]. Common probiotics include *Lactobacillus*, *Bifidobacterium*, *Lactococcus*, *Bacillus*, *Streptococcus*, and yeasts such as *Candida* and *Saccharomyces* [18]. Probiotic strains derived from animals, such as those isolated from the gastrointestinal tract of mammals, are gaining significant attention due to their potential to modulate both local and systemic immune responses. These animal-derived probiotic strains contribute to immune health by influencing the gut-associated lymphoid tissue (GALT), improving intestinal barrier integrity, and enhancing the production of specific immune factors like immunoglobulins and cytokines [19]. In a recent study [20], a novel *Limosilactobacillus reuteri* (previously known as Lactobacillus reuteri) strain, RGW1, isolated from the feces of healthy calves, was characterized for its probiotic properties, including its immunomodulatory effects. Administration of RGW1 led to a marked increase in the levels of anti-inflammatory cytokines, including TGF-β and IL-10. Studies on BALB/c mice have shown that animal-derived probiotic strains have a significant impact on the immune system. *Lactobacillus gasseri* SBT2055 (LG2055), administered for 5 weeks, increased IgA production and the number of IgA+ patches in Peyer’s patches and the lamina propria [21]. Similarly, a mixture of the species *L. acidophilus*, *L. casei*, *L. reuteri*, *B. bifidum*, and *Streptococcus thermophilus*, given for 20 days, led to an increase in regulatory T cells (CD4+ Foxp3+) while reducing the population of Th1, Th2, and Th17 cells, suggesting a potential immunomodulatory effect [22]. The immunomodulatory function of probiotics is illustrated in Figure 2.

Pet ownership has increasingly evolved, with many owners now treating their pets as integral members of the family, colleagues, and even friends [23]. The gastrointestinal system plays a vital role in the overall health of animals, hosting a complex and diverse microbial community. A balanced and healthy gut microbiome is crucial for influencing overall host physiology, well-being, nutrient absorption, metabolism, and the host’s immune functions. Probiotics, defined as living microorganisms that provide health benefits to the host when consumed in adequate amounts, are gaining popularity in pet care [24].

As more and more studies concerning microbiome and host intricate relationships emerge, both in animals and humans, probiotic science gains significant recognition. The scientists need to meet the clinicians and answer some of the most frequently asked questions: What product is the most suitable for the patient? In general, the key factors a clinical specialist should consider when selecting a probiotic product for their patient include the documented efficacy of a specific probiotic strain for a particular species, the therapeutic dose, exclusion of contraindications, and consideration of potential interactions between the probiotic preparation and the host organism.

Among the diverse intestinal microorganisms, those that are selected as probiotics are typically those that can positively influence the host by promoting a balanced intestinal microbiota. These include species from genera such as *Bifidobacterium*, *Enterococcus,* and the family *Lactobacillaceae* [25].

### 2.1. Probiotics Implications on Human Health with Clinical Application in Certain Diseases

#### 2.1.1. Probiotic Therapy Alongside Antibiotics Administration

Research carried out on human medicine proves that probiotics show potential in decolonizing multidrug-resistant (MDR) pathogens from the gut (e.g., *Escherichia coli*, MDR Gram-negative *Bacillus*, *Staphylococcus aureus*, *Clostridium difficile*, and *Helicobacter pylori*), making them a viable alternative to antibiotics [26], which are known to disrupt gut microbiota and promote antibiotic resistance [27]. Probiotics and prebiotics, either alone or in combination with antibiotics, may help restore microbial diversity and improve long-term health outcomes. Findings from a meta-analysis [28] suggest that probiotics can effectively decolonize pathogens in the gut, regardless of bacterial type, making them a promising alternative to conventional antibiotic treatments. Subgroup analysis revealed variations in the effectiveness of probiotics in pathogen decolonization. *Saccharomyces boulardii* demonstrated the highest efficacy, particularly in clearing *Clostridioides difficile* (CDI) during or after antibiotic treatment, followed by other probiotics like *E. coli* Nissle 1917 (EcN), *Enterococcus faecium*, and non-toxigenic *C. difficile* [29]. The success rate of probiotics varied depending on the specific pathogen, with notable effects on CDI, multidrug-resistant *Enterobacteriaceae*, and vancomycin-resistant *Enterococci* (VRE). These differences suggest that the ability of probiotics to eliminate pathogens is influenced by factors such as competition for adhesion sites, production of antimicrobial compounds, immune modulation, and gut barrier reinforcement [30]. However, probiotic dosage, ranging from 10^9^ to 10^10^ colony-forming units (CFU)/day, and study location did not significantly impact the results. While a minimum dose of 10^9^ CFU/day appears necessary for beneficial effects, further clinical studies are required to standardize probiotic formulations, assess strain combinations, and optimize their decolonization potential [31].

#### 2.1.2. Probiotics in *Helicobacter pylori* (*H. pylori*) Infection

Probiotics have emerged as a potential adjunct therapy for various diseases, including *Helicobacter pylori* (*H. pylori*) infection, autoimmune disorders, hypertension, inflammatory bowel disease (IBD), oral candidiasis, autism spectrum disorder (ASD), migraine, and diabetes [32]. *H. pylori*, a Gram-negative flagellated bacterium colonizing the stomach epithelium, has shown increasing resistance to standard triple therapy (STT) due to its impact on natural flora and adverse side effects [33]. Probiotics, particularly from the family *Lactobacillaceae*, exhibit immunomodulatory and antimicrobial properties that may inhibit *H. pylori* infection and enhance eradication rates while reducing gastrointestinal inflammation. Recent studies have explored genetically modified probiotics, such as *Lactococcus lactis* NZ9000 producing *H. pylori* lipoprotein Lpp20 and *Bacillus subtilis* spores expressing *H. pylori* urease B protein, demonstrating promising immune responses [34].

Various probiotic microorganisms, such as those from species *Enterococcus faecium*, *Lactobacillus helveticus*, and *Lacticaseibacillus rhamnosus* (previously known as *Lactobacillus rhamnosus*), have demonstrated reductions in total cholesterol (TC), Low-Density Lipoprotein Cholesterol, and Non-High-Density Lipoprotein Cholesterol, particularly in individuals with hypercholesterolemia [35]. The mechanisms behind these effects include cholesterol assimilation, bile salt deconjugation, production of SCFAs, and modulation of gut microbiota [36].

#### 2.1.3. Probiotics in Metabolic Health

Additionally, probiotics have shown potential in improving metabolic health in patients with type 2 diabetes mellitus (T2DM) and dyslipidemia [37]. Studies suggest that multistrain probiotic formulations can reduce endotoxin levels, improve glycemic parameters, and enhance lipid profiles. Probiotic strains from species *Lactiplantibacillus plantarum* (previously known as *Lactobacillus plantarum*) have also been linked to beneficial changes in gut microbiota composition and SCFA production, which may contribute to obesity prevention and metabolic regulation [38]. Another study shows that probiotics offer benefits in both type 1 and type 2 diabetes (T2DM). In type 1 diabetes, specific strains like *Bifidobacterium longum* subsp. *longum* JCM 1217^T^, *B. longum* subsp. *infantis* 157F (BF)^13^, *B. longum* subsp. *infantis* JCM 1222*^T^*, *L. brevis* KLDS 1.0727, *L. brevis* KLDS 1.0373, *Lactiplantibacillus plantarum* TN627, and *L. fermentum* MTCC, as well as species like *L.acidophilus*, *Lacticaseibacillus casei*, and *L. delbrueckii* subsp. *bulgaricus*, modulate immune responses, reduce pancreatic inflammation, and delay beta-cell destruction [39]. In T2DM, probiotic species such as *Lactiplantibacillus plantarum*, Bifidobacterium lactis, *Lacticaseibacillus rhamnosus*, and *L. gasseri* influence glucose metabolism, lipid homeostasis, and gut microbiota composition, contributing to improved insulin sensitivity, reduced oxidative stress, and lower systemic inflammation [40]. Despite promising findings, some studies report inconsistent results, highlighting the need for further long-term clinical trials to confirm the efficacy and durability of probiotics in managing cholesterol and metabolic disorders [41].

#### 2.1.4. Probiotics in IBD

Probiotics may play a significant role in IBD by inhibiting pathogenic bacteria, thereby protecting the intestinal cells [42]. They enhance the intestinal barrier by stimulating mucus production and antimicrobial peptide release. Additionally, probiotics modify the mucosal immune system, regulating inflammatory responses. By inducing T cell apoptosis, they increase the production of anti-inflammatory cytokines, such as IL-10 and TGF-B, while reducing pro-inflammatory cytokines like TNF-a, IFN-y, and IL-8, helping to manage IBD effectively [43]. In IBD, probiotic strains such as *Lacticaseibacillus paracasei* L74 and the species *Streptococcus salivarius* suppress NF-κB activation and inflammatory cytokine production, and *Lactiplantibacillus plantarum* Lp91 reduces tumor necrosis factor-alpha (TNF-α) and cyclooxygenase-2 (COX-2) expression, promoting intestinal integrity [44].

#### 2.1.5. Probiotics in Gastrointestinal Integrity

*Lactobacillaceae* contribute to gut health by producing lactic acid, which triggers hypoxia-inducible factor (HIF)-2α signaling, reinforcing intestinal barrier integrity. This process was linked to a significant reduction in *Vibrio cholerae* in neonatal mice, suggesting the protective role of lactic acid-producing probiotics [45]. In premature infants, supplementation with *Bifidobacterium bifidum* NCDO 2203 and *Lactobacillus acidophilus* NCDO 1748 resulted in increased fecal acetate and lactate, effectively lowering intestinal pH and restricting the growth of opportunistic pathogens such as *Klebsiella*, *Escherichia*, and *Enterobacter* [46]. Furthermore, EcN has been reported to inhibit biofilm formation of *Pseudomonas aeruginosa* and disrupt mature biofilms, thereby reducing colonization by enterohemorrhagic *Escherichia coli* (EHEC) [47].

#### 2.1.6. Potential Anticarcinogenic Properties of Probiotics

Probiotics exhibit potential anticarcinogenic properties through various mechanisms, including modification of the intestinal microbiota, production of beneficial metabolites, like SCFAs and conjugated linoleic acids (CLAs), and the inhibition of cancer cell growth. They induce apoptosis in cancer cells, e.g., gastric, colonic, and myeloid leukemia cells, modulate mutagenic factors, and enhance immune responses. Many researchers indicate a significant antiproliferative role and/or induction of apoptosis mus musculus colon carcinoma (HGC-27) and human colonic cancer cells (Caco-2, DLD-1, HT-29), and also lowering the level of IL–8 via the strain *Lacticaseibacillus rhamnosus* GG [48,49,50,51,52].

In addition, scientists’ reports indicate the effectiveness of probiotic microorganisms (e.g., Bacillus: polyfermenticus, subtilis; Bifidobacterium: lactis, adolescentis; *Clostridium butyricum*; *Enterococcus faecium*; Lactobacillaceae: *L. acidophilus*, *L. casei*, *L. fermentum*, *L. delbrueckii*, *L. helveticus*, *L. paracasei*, *L. pentosus*, *L. plantarum*, *L. salivarius*; *Lactococcus lactis*; *Pediococcus pentosaceus*, *Propionibacterium acidopropionici*, and *Streptococcus thermophilus*) in reducing proliferation and/or induction of apoptosis human colonic cancer cells such as Caco-2, HT-29, SW1116, HCT116, SW480, DLD-1, LoVo [53].

Moreover, *Lactobacillus acidophilus* CL1285 and *Lacticaseibacillus casei* LBC80R (in the presence of 5-FU) induced apoptosis in human colorectal cells (LS513), while *Lactobacillus acidophilus* SNUL, *Lacticaseibacillus casei* YIT9029, and *Bifidobacterium longum* HY8001 suppressed proliferation of human colorectal (SNUC2A) and gastric carcinoma cells (SNU1) [54]. Probiotics improve intestinal barrier function, degrade carcinogenic compounds, and combat dysbiosis, which is linked to colorectal cancer development. Pathogenic bacteria such as *Bacteroides fragilis* and *Clostridium* spp. contribute to inflammation and tumor progression, while probiotics restore balance by outcompeting harmful bacteria and promoting protective biofilms [55]. Microbial metabolism, particularly the activity of enzymes like azoreductase, β-glucuronidase, and nitrate reductase, can convert dietary components and bile salts into carcinogenic compounds. By modulating these metabolic pathways, probiotics can inhibit harmful enzyme activity, thereby reducing cancer risk. Certain species, like *Lactobacillus acidophilus*, have been shown to lower the activity of these enzymes [56]. Lactic acid bacteria (LAB) contribute to intestinal health by producing organic acids such as lactic and acetic acid, which lower intestinal pH and disrupt pathogenic bacteria. Probiotics also produce bacteriocins with bactericidal properties [57]. SCFAs, particularly butyrate, play a significant role in cancer prevention by regulating inflammation, apoptosis, and cell cycle progression. Butyrate inhibits inflammatory cytokine production, suppresses COX-2 activity, and induces epigenetic changes that favor apoptosis in cancer cells [58]. Conjugated linoleic acids produced by probiotics, such as *Streptococcus thermophilus*: Strain BT01, *Bifidobacterium breve*: Strain BB02, *Bifidobacterium longum*: Strain BL03, *Bifidobacterium infantis*: Strain BI04, *Lactobacillus acidophilus*: Strain BA05, *Lactiplantibacillus plantarum*: Strain BP06, *Lacticaseibacillus paracasei*: Strain BP07, and *Lactobacillus delbrueckii* subsp. *bulgaricus*: Strain BD08, also have anticancer effects, conferred by regulating apoptosis-related genes and suppressing eicosanoid production, which is linked to colon cancer progression [59].

#### 2.1.7. Influence of Probiotics on the Central Nervous System

The microbiome significantly influences the central nervous system (CNS) through a bidirectional communication pathway known as the gut–brain axis (GBA). This interaction occurs via microbial metabolites, which can cross the blood–brain barrier (BBB) and the vagus nerve, modulating the hypothalamic-pituitary-adrenal (HPA) axis and immune responses [60]. Conversely, the brain also impacts gut function by regulating secretion, motility, and permeability, thereby affecting the microbiota. Serotonin, a key signaling molecule, plays an essential role in both the CNS and the enteric nervous system (ENS) within the GBA [61]. Probiotic bacteria influence the CNS through three mechanisms: the production of neuroactive substances, such as neurotransmitters and their precursors, that affect emotions and behavior; the interaction of SCFAs and secondary bile acids with host cells to regulate signaling molecule production; and the activation of signaling molecules through bacterial enzymatic deconjugation. These mechanisms underscore the complex relationship between the microbiome and brain function [62]. The gut microbiota plays a crucial role in maintaining brain health, with imbalances linked to neurodegenerative diseases such as Alzheimer’s disease (AD), Parkinson’s disease (PD), and Huntington’s disease (HD) [63]. In AD, an altered microbiota, characterized by the overgrowth of pro-inflammatory bacteria like *Proteobacteria* and *Escherichia*/*Shigella*, contributes to BBB disruption and disease progression [64]. This imbalance enhances inflammation, a central feature of AD. Certain bacterial infections, such as *Helicobacter pylori*, *Borrelia burgdorferi*, and *Chlamydia pneumoniae*, have also been linked to amyloid-β accumulation and tau phosphorylation, key markers of AD [65]. In PD, microbial disruption, including small intestinal bacterial overgrowth and *Helicobacter pylori* infection, increases gut permeability and motor dysfunction, with a reduction in beneficial bacteria such as *Roseburia* [66]. In HD, a genetic disorder caused by the overexpression of the huntingtin gene, dysbiosis contributes to excessive hydrogen sulfide production and cytokine dysregulation, worsening neurodegenerative symptoms [67]. Research has been carried out on the effects of various probiotic strains on inflammatory cytokines and reactive oxygen species (ROS) production in Parkinson’s disease patients. The results indicated that strains such as *Ligilactobacillus salivarius* LS01 and the species *Lactobacillus acidophilus* significantly reduced pro-inflammatory cytokines and increased anti-inflammatory cytokines, suggesting potential therapeutic benefits [68,69].

#### 2.1.8. Probiotic Preparation Application in Skin Diseases and Wound Healing

Several probiotic strains have been recognized for their positive impact on skin health, particularly in managing various skin conditions. Notable probiotic species include *Lactiplantibacillus plantarum*, *Lactobacillus acidophilus*, *Bifidobacterium longum*, and *Streptococcus thermophilus*. These microbes are known to inhibit the release of pro-inflammatory cytokines, block inflammatory mediators, and support the restoration of skin barrier function [70,71]. These probiotics have been utilized both topically and orally in the treatment of common dermatological conditions, such as acne, atopic dermatitis, and rosacea. Their use helps restore the balance of the skin microbiota, reduces skin inflammation, and improves overall skin health. Studies have investigated the effects of both topical and oral probiotics on skin health. Topical probiotics have demonstrated significant benefits in improving skin hydration and barrier function [72]. Moreover, topical probiotics have shown positive effects in managing various inflammatory skin conditions, including acne, rosacea, and psoriasis, and they also appear to support wound healing [73]. On the other hand, oral probiotics primarily work by modulating the gut microbiota, which indirectly benefits skin health. These probiotics can enhance gut barrier function and reduce systemic inflammation, ultimately improving skin conditions [74]. While topical probiotics tend to produce faster, localized improvements, oral probiotics have more systemic effects, suggesting that both methods may have complementary roles in skin health management. Further studies are needed to directly compare the efficacy of these two forms of probiotic administration using the same strains and to explore any potential synergistic effects between them. One of the main challenges for topical probiotics is their survival under the harsh conditions of the skin, such as low moisture, acidic pH, and immune defenses [75]. Certain strains, such as *Lactiplantibacillus plantarum* ATCC 10241, have shown good survival rates, especially in moist environments like wounds, making them ideal candidates for targeted therapeutic applications [76].

Topical probiotics have garnered scientific interest for their potential to enhance wound healing and prevent inflammation. A study demonstrated that treating burn wounds with *Saccharomyces cerevisiae* MYA-796 resulted in accelerated healing, including increased expression of collagen type 1 and growth factor beta 1 (TGF-β1), as well as improved biomechanical characteristics of the healing skin [77]. Probiotics such as *Lactobacillus acidophilus* CL1285 and *Lacticaseibacillus casei* LBC80R have also shown promise in inhibiting Methicillin-resistant *Staphylococcus aureus* (MRSA), a common wound pathogen. In one study, these probiotics eliminated MRSA growth by 99% after 24 h of incubation. Moreover, the species *Lactobacillus reuteri* and strain *Lacticaseibacillus rhamnosus* GG were found to protect epidermal keratinocytes from *S. aureus*-induced cell death by preventing pathogen adhesion, with the species *L. reuteri* showing superior protection compared to *L. rhamnosus’s* strains. These effects are believed to result from the exclusion of S. aureus from integrin binding sites on keratinocytes [78].

#### 2.1.9. Probiotics and the Immune System

Allergic contact dermatitis (ACD), commonly referred to as eczema, occurs when the skin comes into contact with an allergenic substance, triggering an allergic response. Symptoms include skin inflammation, itching, dryness, and blisters, with the immune response primarily regulated by CD4+ T cells. Both pro- and prebiotics have demonstrated preventive effects on ACD and have been shown to help mediate its symptoms [79]. *Lacticaseibacillus casei*. has been found to reduce skin inflammation through multiple mechanisms, including the inhibition of INF-γ, a cytokine involved in the production of CD8+ effector T cells. Additionally, *L. casei* may promote regulatory CD4+ T cells, further enhancing its anti-inflammatory effects. The species has also been shown to stimulate the production of IL-10 by activating CD4+ CD25+ T regulatory cells (Tregs), which support its role in controlling skin inflammation [80]. EcN is another probiotic that has demonstrated the ability to prevent ACD by increasing the number of Foxp3+ cells, which play a role in suppressing lymphocyte antigen priming [81].

#### 2.1.10. Probiotics in Urinary Tract Infections

Urinary tract infections (UTIs) are often caused by uropathogenic *E. coli* (UPEC). Vaginal probiotics, particularly those containing lactobacilli, could potentially reduce these UPEC reservoirs, thereby decreasing the recurrence of UTIs (rUTIs). *Lactobacillus crispatus* is known for its ability to maintain a healthy vaginal microbiome by reducing pathogen abundance, with *L. crispatus* CTV-05 being an active strain in Lactin-V, a product currently in clinical trials for bacterial vaginosis treatment [82,83]. Recent advancements also include the potential of intravesical delivery of lactobacilli for treating neurogenic lower urinary tract dysfunction. Preliminary studies have shown that intravesical *L. rhamnosus* GG is safe and well tolerated, though its effectiveness in reducing UTI occurrence remains inconclusive [84]. For UTIs, various probiotics have shown positive results, particularly *Lacticaseibacillus rhamnosus* GR-1 and *Lactobacillus reuteri* B-54 when administered vaginally [85,86]. Oerlemans et al. [87] conducted a study on 20 women with vulvovaginal candidiasis (VVC), investigating the effects of a probiotic formulation containing *Lactobacillus pentosus* KCA1, *Lactiplantibacillus plantarum* WCFS1, and *Lacticaseibacillus rhamnosus* GG. The probiotic was administered in doses ranging from 2.5 × 10^9^ to 2.5 × 10^10^ CFU/day for 1.5 weeks. The results indicated that 45% of the women experienced restoration of the vaginal microbiota with the probiotic treatment. However, the remaining 55% required rescue medication, specifically fluconazole.

#### 2.1.11. Probiotics Usage in Hypertension Management

In hypertension management, probiotics contribute to gut microbiota homeostasis, improve intestinal barrier integrity, and lower systemic inflammation. Specific probiotic species, including *Lactobacillus helveticus* and *Saccharomyces cerevisiae*, produce bioactive peptides with angiotensin-converting enzyme (ACE) inhibitory properties, mimicking the effects of ACE inhibitors. The consumption of fermented milk products containing *L. casei* strain Shirota (LcS) has been linked to reduced hypertension risk, attributed to its polysaccharide-glycopeptide complex promoting prostaglandin I2 synthesis and reducing vascular resistance [88].

#### 2.1.12. Probiotics in Maintaining Oral Health

Some bacterial species also contribute to oral health, particularly in reducing *Candida* colonization in denture wearers. *Lacticaseibacillus rhamnosus* and *Limosilactobacillus reuteri* have demonstrated antifungal activity, with probiotic formulations containing *Bifidobacterium longum*, *Lactobacillus bulgaricus*, and *Streptococcus thermophilus* showing superior efficacy when combined with antifungal agents like nystatin. This suggests that probiotic-based interventions may help prevent oral candidiasis in immunocompromised individuals and elderly populations [89].

### 2.2. Health Benefits of Probiotic Usage in Companion Animals and Viable Strains Used in Specific Conditions

In recent years, the application of probiotics in pets, particularly dogs and cats, has been explored with promising outcomes. Research indicates that probiotics can modulate the immune system, enhance gut health, and protect against pathogenic bacteria in companion animals [90]. The consumption of probiotics offers several health advantages, including the prevention of diarrhea, the maintenance of a stable and healthy gastrointestinal microbiome, and support in managing mild enteropathies as well as small intestinal bacterial overgrowth [91].

Shelter animals, prone to diarrhea due to stress and dietary changes, have shown species-specific responses to probiotics; *E. faecium* SF68 reduced diarrhea in shelter cats but not in dogs [92]. Furthermore, probiotics have been investigated for dietary allergies and Helicobacter infections in dogs, especially specific strains from the species *Lactobacillus acidophilus*, *Lactobacillus reuteri*, and *Lactobacillus johnsonii*, improving gut health and clinical symptoms [93]. Given the effectiveness of *Lactobacillus casei* DN-114 001 in eradicating *Helicobacter* infections in pediatric patients, similar therapeutic applications in veterinary medicine may be possible [94]. In feline studies, *Lactobacillus acidophilus* DSM13241 supplementation over 4.5 weeks resulted in increased lactobacilli levels, reduced *Enterococcus faecalis* and *Clostridium difficile*, lowered fecal pH, and decreased endotoxin levels in the blood, suggesting a strengthened immune response [95]. Probiotics have been increasingly utilized in veterinary medicine, particularly in the management and prevention of gastrointestinal disorders in both dogs and cats. Several studies have investigated the efficacy of specific probiotic strains, each demonstrating a variety of health benefits in animals, as presented in Table 1.

Probiotic supplementation in cats has demonstrated various health benefits, particularly in improving gut health and managing gastrointestinal and respiratory conditions. For instance, a combination of *Saccharomyces boulardii* (1 × 10^10^ CFU/kg) and *Pediococcus acidilactici* (1.25 × 10^10^ CFU/kg) was shown to modulate gut microbiota, enhance SCFA production, reduce inflammation, and promote the settlement of beneficial bacteria such as *Lactobacillaceae* and *Bacillus* species in 12 healthy cats [109]. Similarly, *Enterococcus faecium* strain SF68 (5 × 10^8^ CFU/day) reduced the prevalence of diseases associated with chronic feline herpesvirus type 1 (FHV-1) infections in 12 cats [110]. Probiotics have also been beneficial for managing chronic gastrointestinal conditions. For example, a mixture of *Streptococcus thermophilus* DSM32245, *Lactobacillus acidophilus* DSM32241, *Lactiplantibacillus plantarum* DSM32244, *Lacticaseibacillus casei* DSM32243, *Lactobacillus helveticus* DSM32242, *Lactobacillus brevis* DSM27961, *Bifidobacterium lactis* DSM32246, and *Bifidobacterium lactis* DSM32247 (2 × 10^11^ lyophilized bacteria per 5 kg body weight) significantly improved clinical symptoms of constipation and idiopathic megacolon in seven cats [111]. In cats suffering from diarrhea, *Enterococcus faecium* SF68 (2.1 × 10^9^ CFU/day) led to a reduction in diarrhea rates across a study group of 217 cats [112]. Additionally, *Bacillus subtilis* SC06 and *Bacillus coagulans* B10 (3 × 10^9^ CFU/kg) improved digestion, antioxidant capacity, and weight gain in 20 healthy cats, while *Bacillus licheniformis* (1.1 mg/kg) alleviated chronic diarrhea in 8 cats [113]. Furthermore, *Lactobacillus* strains such as *L. acidophilus* CECT 4529 (5 × 10^9^ CFU/kg) and *L. reuteri* NBF 2 DSM 32264 (5 × 10^9^ CFU/kg) improved fecal quality and increased beneficial *Lactobacillaceae* populations in 10 and 12 healthy cats, respectively [114].

The benefits of using probiotics in the treatment of kidney diseases in dogs have also been described. Administration of VSL#3, a probiotic formulation containing *Lacticaseibacillus casei*, *L. plantarum*, *L. acidophilus*, *L. delbrueckii* subsp. *bulgaricus*, *Bifidobacterium longum*, *B. breve*, *B. infantis*, and *Streptococcus salivarius* subsp*. thermophilus* (112 to 225 × 10^9^ CFU/10 kg body weight daily for 60 days), significantly enhanced the glomerular filtration rate (GFR) in dogs diagnosed with chronic kidney disease. The probiotic-treated group exhibited improved renal function compared to both baseline levels and the control group [115]. Chronic kidney disease (CKD) is prevalent among felines, leading to the accumulation of nitrogenous waste products, such as urea, which can be detrimental to health [116]. Recently, a synbiotic supplement was introduced to mitigate uremic toxins in cats with CKD. It combines specific bacterial strains from the species *Streptococcus thermophilus*, *Lactobacillus acidophilus*, and *Bifidobacterium longum*, aiming to metabolize urea and other toxins within the gut, thereby reducing their systemic absorption (Vetoquinol, Fort Worth, TX, USA). The proposed mechanism, termed “enteric dialysis”, involves these probiotics utilizing urea in the intestines, potentially lowering blood urea nitrogen levels. However, clinical evaluations of the product’s efficacy have yielded mixed outcomes [117]. A study by Rishniw and Wynn [118] reported that the method of delivering certain synbiotic products to cats with CKD (sprinkling onto food) significantly reduces the potential of altering azotemia. Another study evaluating a commercial probiotic supplement, *Enterococcus faecium* SF68, in cats with CKD over an 8-week period found no significant changes in the gut microbiome or serum concentrations of indoxyl sulfate (IS) and p-cresyl sulfate (pCS), two uremic toxins [119]. *Lactobacillaceae* supplementation may influence gut microbiota composition and metabolic pathways, leading to potential benefits in CKD management. A feline study examining the effects of *Lactiplantibacillus plantarum* subsp*. plantarum* MFM 30 − 3 and *Lacticaseibacillus paracasei* subsp. *paracasei* MFM 18 intervention in cats with stage 2–3 CKD demonstrated significant changes in microbial composition and serum metabolites, highlighting mechanisms by which probiotics may modulate CKD progression [120].

In a study involving 35 dogs without a history of recurrent urinary tract infections (UTIs) [121], the results suggested that oral probiotics could potentially influence the vaginal microbiota in dogs, although further investigation is needed to understand the full effects and the optimal probiotic strains for canine health. The oral probiotic used contained *Lactobacillaceae*, *Bifidobacterium*, and *Bacillus* species. A case report from India suggests a positive impact of probiotic combination treatment in managing Feline Idiopathic Cystitis (FIC) [122]. The probiotic combination administered included the species *Lacticaseibacillus casei* (4 × 10^8^ CFU), *Lacticaseibacillus rhamnosus* (3 × 10^8^ CFU), *Lactobacillus acidophilus* (5 × 10^7^ CFU), *Lactobacillus bulgaricus* (1 × 10^7^ CFU), *Bifidobacterium infantis* (4 × 10^7^ CFU), *Bifidobacterium breve* (5 × 10^7^ CFU), and *Streptococcus thermophilus* (1 × 10^8^ CFU). The exact probiotic strains have not been stated. After the initial treatment phase, medication was continued orally for an additional 5 days, including pipemidic acid, diazepam, neurotropic vitamins, and doxycycline. *Lactobacillus* species demonstrated antimicrobial properties by producing H_2_O_2_ [123], which inhibits the growth of uropathogenic *E. coli* (UPEC), *Salmonella* sp., and *Helicobacter pylori.* Furthermore, probiotics contributed to immune modulation, significantly reducing IL-6, IL-8, and lactic acid dehydrogenase levels, which in turn exerted anti-inflammatory effects [124].

Additionally, probiotics have been associated with enhanced immune responses, providing a protective effect against various diseases [125]. Probiotics exert immunomodulatory effects by promoting T-cell differentiation, regulating the balance of pro- and anti-inflammatory cytokines, and enhancing secretory IgA (sIgA) production [126]. The impact of *Enterococcus faecium* SF68 supplementation on immune responses following administration of a multivalent vaccine was assessed in kittens. *E. faecium* SF68 was detected in the feces of seven out of nine cats. Notably, the percentage of CD4+ lymphocytes was significantly increased in the treatment group, although no significant differences were observed in other immune parameters between the groups [99].

Probiotic therapy seems to engage in modulating the behavior and mental health of companion animals, particularly dogs and cats. The GBA, as described before, is a bidirectional communication network between the gastrointestinal tract and the central nervous system, playing a pivotal role in this interaction. A study conducted in dogs found that supplementation with *Saccharomyces boulardii* (1 × 10^9^ CFU per kg of feed) led to a reduction in fecal calprotectin, immunoglobulin A (IgA), and cortisol levels, suggesting that *S. boulardii* may contribute to alleviating intestinal inflammation and decreasing stress hormone production [127]. Additionally, a 14-day supplementation with *Lactiplantibacillus plantarum* PS128 seemed to stabilize behaviors associated with aggression and separation anxiety. Plasma 5-HT turnover ratio was found to decrease after supplementation, particularly in dogs with separation anxiety. This suggests that 5-HT may play a role in the GBA, as the slower breakdown of 5-HT into its metabolites leads to higher availability of this neurotransmitter in the system [128]. Probiotic interventions have gained attention for their potential in alleviating anxiety-related behaviors in dogs. One study performed by Purina^®^ researchers investigated the effects of *Bifidobacterium longum* (BL999) on anxious Labrador Retrievers. The results showed significant improvements in both behavioral and physiological markers, including heart rate and salivary cortisol, suggesting the potential of BL999 to reduce anxiety. In a separate double-blind, placebo-controlled trial [129], Relaxigen Pet dog^®^, a nutraceutical supplement containing prebiotics, probiotics (*Lacticaseibacillus reuteri*), postbiotics, and neuroprotective compounds, was evaluated in anxious dogs. The treated dogs exhibited a reduction in microbials like *Bacteroides* and *Lactobacillus*, along with a decrease in anxiety-like behaviors. However, a recent study showed that there were no major changes in the gut microbiota of dogs with phobic behavioral disorder, except for an increase in *Lactobacillus*, which is known for its probiotic properties [130]. Chronic treatment with *Lacticaseibacillus rhamnosus* JB—1 has been shown to impact anxiety and depression-related behaviors by modulating GABA receptor mRNA expression in specific brain regions [131]. Although the factors behind the increased abundance of *Lactobacillus* in phobic dogs are unclear, the authors speculated that the presence of this psychobiotic could contribute to the development of phobic behavioral traits.

A study conducted by Barthe et al. (2023) assessed the effects of topical probiotic bacteria on canine progenitor epidermal keratinocytes (CPEK) [132]. Non-formulated probiotics were applied at concentrations of 75, 750, and 7500 CFU/mL for 24 h. At 7500 CFU/mL, only 2% of the probiotic bacteria were dead, while at lower concentrations (750 and 75 CFU/mL), 81% and 84% of those bacterial agents were dead, respectively. This indicated that higher concentrations maintained more viable probiotic products. In a scratch wound assay, non-formulated probiotics enhanced CPEK cell migration in a dose-dependent manner, with 16% improvement at 750,000 CFU/mL. The vehicle used in the formulation also increased migration by up to 14%. However, formulated probiotics provided the most significant wound healing enhancement, increasing migration by 20% at the highest concentration.

Human and canine microbiomes seem to be similar, with canines posing as models for studying the human microbiome [133]. Thus, some probiotic products designed for humans may be utilized in canine medicine, provided that the probiotic strain is adequate and examined for canines and the amount of the product (CFU/day) corresponds to those tested for the species.

It is important to note that while the current evidence is promising, research on the use of probiotics in companion animals is still limited.

### 2.3. Probiotic Therapy in the Animal Production Sector

In recent years, there has been increasing scientific and commercial interest in the incorporation of probiotics into animal feed as a strategy for preventing or managing various animal diseases. This approach is gaining traction as a viable alternative to the use of growth-promoting antibiotics, which can lead to undesirable side effects and adverse reactions in animals [134]. The use of probiotics in animal production has become a promising strategy to improve growth, feed efficiency, health, and meat quality across various livestock species, including poultry, swine, and cattle [135].

Probiotic supplementation has been shown to enhance microbial diversity, improve gut health, and positively affect nutrient digestion and immunity [136]. Species like *Lactiplantibacillus plantarum* and *Bacillus subtilis* are among the most widely used probiotics in livestock, promoting beneficial metabolic processes such as short-chain fatty acid production and improving immune responses. Studies have demonstrated the efficacy of specific probiotic strains, such as *L. plantarum* PFM 105, in increasing short-chain fatty acid production compared to antibiotics, while *B. subtilis* strains have been found to reduce the abundance of harmful bacteria in the gastrointestinal tract of swine and poultry, contributing to enhanced health outcomes [137]. However, the effectiveness of probiotics can vary depending on factors like dosage, dietary formulation, and the microbiota composition of the host [138]. Research also suggests that probiotic treatment may have unintended consequences, such as disrupting the native gut microbiota, which could potentially increase the risk of future diseases [139]. Long-term colonization of probiotic strains is often desirable, but competition between introduced and native microbiota needs to be considered to ensure successful engraftment. Recent findings emphasize the potential of multistrain probiotics, as they may provide a broader ecological range, minimizing competition with resident microorganisms [140]. Available data also suggest a connection between the gut microbiome and neurological changes, influencing feeding behaviors in farm animals, although the underlying mechanisms remain unclear [141]. However, the efficacy of probiotics in comparison to traditional antibiotic growth promoters remains a challenge. Studies indicate that certain probiotic strains may not be as effective as antibiotics or implants in promoting faster growth [142]. The variation in efficacy is influenced by factors such as microbial strain composition, dosage, delivery methods, environmental stress, and the health condition of the animal.

#### 2.3.1. Swine

Studies highlight the positive impact of probiotics such as *Bacillus coagulans* GBI-30, 6086 and the species *Clostridium butyricum* on growth performance and nutrient digestibility in pigs, with *Bacillus* strains enhancing protein consumption and nutritional absorption [143]. Among the various probiotic strains utilized in swine nutrition, those belonging to the genera *Lactobacillus* and *Bacillus* are particularly prominent. A meta-analysis focusing on *Lactobacillus*-based probiotics, including species such as *Lactobacillus delbrueckii*, *Lactobacillus reuteri*, *Lactiplantibacillus plantarum*, and *Lactobacillus acidophilus*, demonstrated improvements in the growth performance and intestinal morphology of piglets [144]. The supplementation of these probiotics was associated with enhanced villus height and a reduced crypt depth in the intestines, indicating better nutrient absorption and gut health [145]. Yet, probiotic efficacy needs to be carefully considered.

A study evaluating the impact of a probiotic containing *Bacillus subtilis* DSM25841 and *Bacillus amyloliquefaciens* DSM25840 on sows and their piglets found that dietary supplementation improved reproductive performance, increased the birth and weaning weights of piglets, and enhanced fecal microbiota composition. Specifically, sows receiving the probiotic exhibited higher average daily feed intake during lactation and reduced body weight loss, while their piglets had higher birth weights and improved growth rates [146].

In swine production, multistrain probiotics play a vital role in enhancing growth performance, feed efficiency, and overall metabolic utilization of nutrients. In piglets, combinations of probiotics, such as *Ligilactobacillus salivarius* ZJ614, *Lactobacillus reuteri ZJ625*, and *Streptococcus salivarius* NBRC13956, have shown positive impacts on blood profiles (hemoglobin and hematocrit, neutrophils, monocytes, lymphocytes, eosinophils, basophils, platelets, total serum protein, albumin, globulin, cholesterol, and glucose) and overall health. Supplementation with such probiotics also significantly increased IgG serum levels, which is crucial for preventing postweaning diarrhea, and reduced the population of enteric bacteria while increasing the population of lactic acid bacteria [147]. Probiotics containing strains from such species as *Bacillus lichenformis*, *Bacillus coagulans*, and *Bacillus subtilis* have been linked to increased weight gain, improved feed efficiency, and reductions in harmful gas emissions like hydrogen sulfide and mercaptans, which are of environmental concern [148]. Additionally, high doses of these probiotics have been shown to increase the digestibility of dry matter, nitrogen, and energy, as well as modulate the microbial populations in the feces, particularly by reducing *E. coli* counts. In reproductive swine, probiotics such as *Enterococcus faecalis* DSM 7134 (species *Clostridium butyricum* and *Bacillus mesentericus*) administered before farrowing have been shown to improve reproductive performance by enhancing the return of sows to estrus and optimizing farrowing outcomes [149]. However, some studies report no effect on the reproductive performance of lactating sows, suggesting that the benefits of probiotics in swine may be strain-specific [150].

There is emerging evidence on the use of probiotics in wound healing in pigs. In one study investigating the effects of topical treatments on three full-thickness skin wounds created on the dorsum of each animal [151], the pigs remained comfortable, with consistent feed intake and no changes in activity or social interaction. By day 15, all wounds had healed with normal progression, contracting to a minimal surface area. No significant difference in wound appearance was observed between the treatment and control groups. The study suggests that topical treatment with *S. boulardii* (no indication of specific strain used in the study) did not significantly alter the bacterial profile or wound healing in terms of surface area reduction, but histological analysis showed typical wound healing features.

The effects of the application of selected probiotic strains in swine are listed in the Table 2.

#### 2.3.2. Poultry

Probiotics have been increasingly utilized in poultry production to enhance growth performance, bolster immune responses, and improve gut health. Specific strains from the species *Lactobacillus acidophilus* have been shown to stimulate cytokine production, thereby enhancing the immune response in broiler chickens. Additionally, strains from the genus *Bacillus*, known for their resilience to high temperatures and acidic pH, are commonly employed in poultry diets. These probiotics have demonstrated efficacy in improving nutrient utilization and maintaining gut health [156]. Furthermore, host-specific probiotics, derived from bacterial strains that have coevolved with poultry, have shown greater potential in providing health benefits compared to non-host-specific strains. This host-specific approach may enhance the colonization and efficacy of probiotics in the avian gut. Research into probiotic multistrain usage in poultry has demonstrated that mixtures containing microorganisms with probiotic potential, such as *Saccharomyces cerevisiae*, *Lactobacillus fermentum*, *Pediococcus acidilactici*, *Lactiplantibacillus plantarum*, and *Enterococcus faecium*, can enhance feed efficiency, growth, and intestinal health in broiler chickens, especially when challenged with *Pasteurella multocida* [157]. A combination of such species: *Enterococcus faecium*, *Lactobacillus acidophilus*, *Lactiplantibacillus plantarum*, and *Bifidobacterium bifidum* improved overall performance in chickens, including enhanced gut structure, reduced lipid peroxidation, and a reduction in *Clostridium* spp. populations [158]. Despite these promising results, the benefits of probiotics in poultry are not always uniform. For example, certain probiotic formulations have shown no effect on broiler breeder performance or cholesterol levels [159].

The effects of the application of selected probiotic strains in poultry are listed in Table 3.

#### 2.3.3. Cattle

Furthermore, probiotics can enhance milk production in dairy cows. For example, supplementation with *Bacillus subtilis* and *Bacillus licheniformis* has increased milk protein and fat content, while *Lactobacillus* strains have been linked to higher milk output [163]. Additionally, probiotics can help reduce the incidence of mastitis in dairy cows [164]. In dairy cows, supplementation with yeast strains, particularly *Saccharomyces cerevisiae*, has been shown to improve ruminal fermentation, leading to increased fiber digestion and milk yield. These yeast probiotics enhance cellulolytic activity and microbial protein synthesis in the rumen, contributing to better nutrient utilization [165]. In beef cattle, the use of lactic acid bacteria such as *Lactobacillus acidophilus* has demonstrated benefits in growth performance and health. For instance, administering *Lactobacillus acidophilus* NP51 at a concentration of 10^9^ CFU per day to steers over a 126-day period resulted in a 37% reduction in *Escherichia coli* O157:H7 shedding, thereby enhancing food safety [166]. In young calves, early-life probiotic supplementation has been linked to enhanced weight gain and disease resistance. Probiotics can modulate the gut microbiota, leading to improved nutrient absorption and immune function, which are critical during the early stages of development [167]. For instance, a multispecies probiotic consisting of *Bifidobacterium bifidum*, *Pediococcus acidilactici*, *Lactobacillus acidophilus*, *Lacticaseibacillus casei*, and *Enterococcus faecium* has been found to reduce the duration of diarrhea in dairy calves while also improving daily weight gain [168]. In buffaloes, a multistrain probiotic mixture containing *Streptococcus faecium*, *Lacticaseibacillus casei*, *Lactobacillus acidophilus*, *Lactobacillus bulgaricus*, *Lactobacillus reuteri*, and *Lactobacillus lactis*, along with *Aspergillus oryzae* and *Saccharomyces cerevisiae*, led to an increased milk yield and improved feed conversion ratio, despite no significant effects on body condition or dry matter intake [169].

Recent research has explored alternative therapies to antibiotics for preventing and treating uterine diseases in cows, with probiotics emerging as a promising option. One of the most commonly studied groups of probiotics is LAB, particularly *Lactobacillus* spp., due to their ability to produce lactic acid, which helps maintain optimal vaginal pH and inhibits the growth of pathogenic bacteria [170]. Various strains of *Lactobacillus* spp., such as *L. rhamnosus*, *L. sakei*, and *Pediococcus acidilactici*, have demonstrated potential for inhibiting pathogens like *E. coli* and *T. pyogenes* in vitro. For instance, *P. acidilactici* was found to produce bacteriocins that could inhibit pathogenic bacteria, while combinations of LAB strains showed enhanced effectiveness in reducing *E. coli* infection and related inflammation [171]. In vivo studies have focused on the application of intravaginal probiotics, demonstrating positive outcomes in reducing the incidence of uterine infections, improving uterine health, and even enhancing reproductive performance [172]. For example, a mixture of *L. sakei*, *P. acidilactici*, and *L. reuteri* administered intravaginally reduced the occurrence of purulent vaginal discharge and improved milk yield in dairy cows [173]. Additionally, intravaginal treatments have been shown to reduce metritis prevalence and improve uterine involution, leading to increased fertility rates [174].

Overall, while probiotics offer numerous benefits in animal husbandry, their effectiveness depends on various factors (age, sex, nutrition, and genetics), requiring careful consideration to maximize their potential.

The effects of the application of selected probiotic strains in cattle are listed in Table 4.

### 2.4. Influence of Probiotics on Bioavailability of Drugs

Probiotics can significantly influence the pharmacokinetics of various drugs by altering their absorption, metabolism, and bioavailability through several mechanisms, including changes in gut microbiota composition, microbial enzyme activity, and intestinal transport processes. For example, *Lactobacillus acidophilus*, *Bifidobacterium lactis*, and *Streptococcus salivarius* increased the activity of azoreductase and enhanced the metabolism of sulfasalazine in Wistar rats [180]. In humans, *L. acidophilus* decreased nitroreductase and azoreductase activity, reducing the toxicity of nitrazepam [181]. Similarly, *B. lactis* increased dopamine levels in human studies, and *L. brevis* enhanced tyrosine decarboxylase activity in vitro. Probiotics can also modulate the bioavailability of antidiabetic drugs such as gliclazide, with *L. acidophilus*, *L. rhamnosus*, and *B. lactis* increasing bioavailability in diabetic rats but decreasing it in healthy rats [182]. In contrast, *Lacticaseibacillus casei* delayed the peak plasma concentration of amiodarone in rats, whereas *E. coli* Nissile 1917 serotype O6:K5:H1 increased amiodarone bioavailability [183]. Additionally, *B. lactis*, *B. longum*, *B. bifidum*, *L. acidophilus*, *L. rhamnosus*, and *S. thermophilus* were shown to reduce toxicity in amlodipine-treated rabbits [184] and enhance bioavailability. Probiotics also play a role in reducing the toxicity of chemotherapy drugs like irinotecan, with *L. plantarum*, *L. casei*, *L. acidophilus*, and *B. longum* reducing β-glucuronidase activity and irinotecan toxicity [185]. Moreover, studies on pain medications, such as indomethacin and paracetamol, suggest that probiotics like *L. casei* CRL 431, *L. paracasei* CNCM I-1518, and *L. reuteri K8* can mitigate drug toxicity and modulate biotransformation pathways [186].

### 2.5. The Role of Probiotics in Digestion and Nutrient Absorption

Probiotics may play a significant role in enhancing digestive processes and nutrient absorption. Recent studies have elucidated various mechanisms through which probiotics influence these physiological functions [187,188].

Probiotics contribute to digestion by modulating the gut microbiota, leading to improved breakdown of dietary components [189]. Certain *Bifidobacterium bifidum* strains, such as *B. bifidum* PRL2010, *B. bifidum* TMC3115, *B. bifidum* UCC2003, and *B. bifidum* JCM1217, produce enzymes that aid in the hydrolysis of complex carbohydrates and proteins, facilitating their assimilation [190]. Additionally, probiotics can stimulate the host’s digestive enzyme activity, further enhancing nutrient breakdown [191]. In animal models, probiotic supplementation has been associated with increased villus height and crypt depth in the small intestine, morphological changes that are indicative of enhanced nutrient absorption capacity. Moreover, probiotics have been shown to improve the expression of nutrient transporters, such as Glucose transporter 2 (GLUT2), thereby facilitating the uptake of glucose and other monosaccharides [192].

Probiotics also affect the bioavailability of various micronutrients through several mechanisms. Certain probiotic species, such as *Lactiplantibacillus plantarum*, *Limosilactobacillus fermentum*, *Bifidobacterium bifidum*, and *Bifidobacterium longum*, can synthesize B vitamins, including folate, vitamin B12, and riboflavin, directly within the gut lumen, contributing to the host’s vitamin pool [192]. Probiotic activity can lower intestinal pH through the production of short-chain fatty acids, enhancing the solubility and absorption of minerals like calcium, iron, and zinc [193]. Some probiotic species, such as *Lactiplantibacillus plantarum*, *Limosilactobacillus fermentum*, *Lactobacillus acidophilus*, and *Bifidobacterium breve*, can degrade phytates and oxalates, compounds that otherwise inhibit mineral absorption, thereby improving the bioavailability of these nutrients [194]. Clinical trials have demonstrated that supplementation with specific probiotic species such as *Lactobacillus helveticus* and *Lacticaseibacillus paracasei* (no particular strains were mentioned in the study) can lead to measurable increases in serum levels of these micronutrients, underscoring their potential role in addressing nutrient deficiencies [191]. Probiotics may also influence gastrointestinal motility, which is crucial for optimal digestion and nutrient absorption. For instance, *Lacticaseibacillus rhamnosus* GG has been shown to enhance gastric emptying and intestinal transit times, thereby facilitating more efficient nutrient assimilation. These effects are thought to be mediated through the modulation of gut hormones and neurotransmitters involved in motility regulation [195].

The integration of probiotics into the diet presents a promising avenue for enhancing digestive health and nutrient absorption. Through various mechanisms—including enzyme production, modulation of gut morphology, vitamin synthesis, and improvement of mineral bioavailability—probiotics can play a pivotal role in optimizing nutritional status. However, the efficacy of probiotic interventions is strain-specific and influenced by factors such as dosage, duration of administration, and individual host characteristics. Further research is warranted to delineate these variables and to establish standardized guidelines for probiotic use in nutritional therapy.

### 2.6. Probiotics as the Connecting Link in the One Health Concept

In the One Health approach, great emphasis is put on the interconnection between humans, animals, and the environment. These factors cannot function properly if one of them is missing or harmed. In this framework, probiotics play a great role as a link connecting all parts, potentially benefiting all three sectors simultaneously. The growing use in veterinary medicine, agriculture, and environmental management positions probiotics as one of the strategies of the One Health concept [196].

The use of probiotics in both human and animal medicine lowers the use of antibiotics, reducing the reliance of treatment on these drugs, therefore addressing one of the most critical One Health issues: antimicrobial resistance [197].

Probiotics used in agriculture enhance biodiversity, reduce the ecological footprint of food production, and, as mentioned above, limit the spread of resistant bacteria and genes into the environment [198].

Probiotics serve as a bridge between human, animal, and environmental health. Their responsible use aligns with One Health goals. As global health challenges become more and more connected in these three sectors, as proven, for example, by the SARS-CoV-2 pandemic, probiotics serve as a possibility of managing all of them by using one solution [199].

## 3. Key Characteristics of New Probiotic Strains Derived from Animals

### 3.1. Tolerance to Environmental Conditions

Newly isolated probiotic strains from animals ought to exhibit key characteristics that determine their survival and efficacy under various environmental conditions. A fundamental aspect of these probiotics is their resilience to harsh conditions, particularly the challenges posed by the gastrointestinal tract (GIT). To function effectively in humans, companion animals, and livestock, probiotic strains must demonstrate tolerance to factors such as low pH, bile salts, enzymatic degradation, and temperature fluctuations [200]. Their ability to thrive in such environments is critical for ensuring their viability and effectiveness when administered.

The successful application of probiotic strains isolated from animals requires optimization of manufacturing processes, ensuring viability during storage and transportation, and resilience through gastrointestinal transit. Each stage presents significant challenges that must be addressed to maintain probiotic efficacy [201]. Scaling up probiotic production introduces variations in cultivation conditions, including pH, medium composition, and gas atmosphere, affecting cell survival and metabolic stability [202]. Large-scale fermentation requires stringent control of homogeneity and holding times to maintain quality [203]. Post-fermentation processing, particularly freeze-drying and spray-drying, imposes osmotic, oxidative, and thermal stresses, potentially leading to membrane damage and viability loss. Freeze-drying risks intracellular ice formation, while spray-drying primarily induces heat stress, affecting membrane integrity [204].

Studies have shown that probiotics derived from livestock, including ruminants, pigs, and poultry, must be rigorously tested for survival under conditions mimicking the GIT environment. These conditions include acidic gastric environments (pH 2–3), bile salt concentrations, and the presence of digestive enzymes, such as pepsin, which challenge microbial survival and activity. Upon ingestion, probiotics encounter salivary enzymes, such as lysozyme, though their impact on viability is minimal due to the transient exposure time [205]. However, the gastric environment presents a major barrier, with acidic pH (0.9–3.0), digestive enzymes, and hydrochloric acid posing significant threats to bacterial survival [206]. Acid stress can lead to intracellular acidification, disruption of membrane integrity, and depletion of ATP due to the reversal of the F1F0-ATPase function [207]. Strains such as *Lacticaseibacillus rhamnosus* GG and those from the species *Saccharomyces boulardii* have been evaluated for their ability to withstand these stressors and demonstrate efficacy in animal models as well as in humans [208]. Probiotic yeasts isolated from animal feces, such as *Kodamaea ohmeri*, *Trichosporon asahii*, *Trichosporon* spp., *Pichia kudriavzevii*, and *Wickerhamomyces anomalus*, have shown promising resistance to low pH and bile salt concentrations. Among these, *W. anomalus* exhibited the highest capacity for agglutination and adherence, demonstrating its ability to grow under acidic and stressful conditions, which enhances its potential as a probiotic in harsh gastrointestinal environments [209]. Furthermore, yeasts isolated from ruminal liquid, including *Magnusiomyces capitatus*, *Candida ethanolica*, *Candida paraugosa*, *Candida rugosa*, and *P. kudriavzevii*, have been shown to effectively reduce pH, accumulate acids, and improve the digestibility of neutral detergent fiber. These findings indicate the survival and functionality of these microorganisms within the ruminal environment, offering potential benefits for ruminant nutrition [210]. Additionally, *Debaryomyces hansenii* has been highlighted for its strong immunomodulatory effects in in vitro studies, likely attributed to its production of polyamines and the presence of β-D-glucan in its cell wall, which contribute to its beneficial immune-stimulating properties [211]. These studies underscore the promising applications of yeasts as probiotics for both gastrointestinal health and animal nutrition.

Upon entering the small intestine, probiotics must withstand bile salts, pancreatic enzymes, and a sudden shift to a more neutral pH (~6.0), which can destabilize membranes and proteins [212]. Bile acids, particularly their conjugated forms, act as biological detergents, disrupting membrane integrity and dissipating the proton motive force, leading to ion leakage, oxidative stress, and potential cell death. However, probiotic strains with bile salt hydrolase (BSH) activity demonstrate enhanced survival by deconjugating bile acids, reducing their toxicity [213]. Species like *Lactobacillus* spp. isolated from ruminants and swine have demonstrated enhanced resistance to digestive enzymes such as pepsin and pancreatin, ensuring their survival and retention of probiotic functionality after GIT transit [214]. Furthermore, biofilm formation—a key adaptive mechanism—enhances the resilience of probiotics by promoting adherence to intestinal mucosal surfaces and increasing resistance to environmental stressors. *Lactobacillus* spp. and *Bifidobacterium* spp., derived from animals, exhibit strong biofilm-forming capabilities, allowing them to establish stable populations in the host’s intestine [215].

To counteract these stressors, newly animal-derived probiotics should employ both innate and adaptive mechanisms. Intrinsic resistance includes cell envelope modifications and metabolic adjustments, while adaptive responses involve changes in membrane composition, upregulation of chaperones, stress-response proteins, and DNA repair enzymes [216]. These strategies collectively enhance survival and functional stability, highlighting the importance of strain-specific selection and formulation techniques in probiotic development.

### 3.2. Production of Antimicrobial Properties and Bioactive Compounds

#### 3.2.1. Bacteriocins

Another essential characteristic of new animal-derived probiotics is their ability to produce antimicrobial compounds that inhibit pathogenic microorganisms within the gut. Bacteriocins are cationic peptides with antimicrobial properties that exert their action primarily through the formation of pores in the target cell membranes, leading to the dissipation of cytosolic contents and subsequent cell death. In addition to their direct antimicrobial activity, bacteriocins are also involved in modulating the host’s native microbiota. This modulation can positively influence host immune responses, contributing to enhanced health outcomes. The ability of bacteriocins to alter microbial community structures and promote beneficial host–microbe interactions highlights their potential as therapeutic agents in preventing or treating infections. Furthermore, the immune-regulatory effects of bacteriocins may enhance the host’s resilience to pathogens, thus supporting overall health-promoting functions [217].

**Figure 3:**
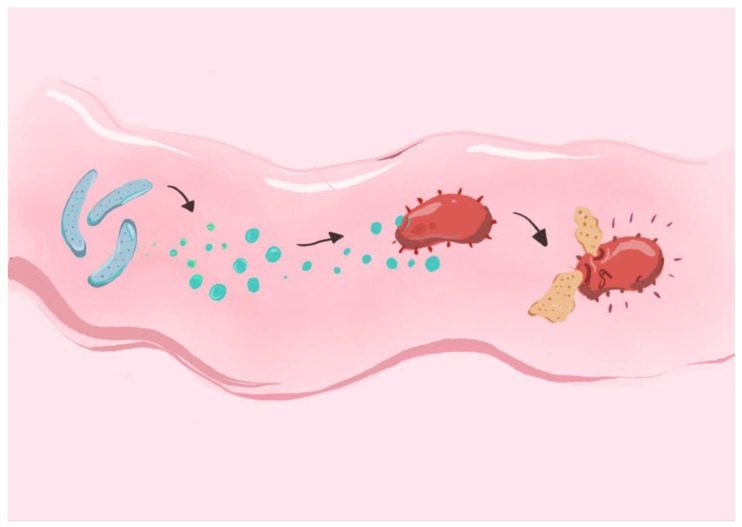
Bacteriocin-mediated effects of probiotic strains on pathogen inhibition: probiotic bacteria, such as those from *Lactobacillus* spp., secrete bacteriocins that act on the pathogenic bacterial cells. By creating pores in the target cell membranes, bacteria-derived antimicrobial compounds lead to the dissipation of cytosolic contents and subsequent cell death.

Strains isolated from pigs and poultry, such as those from *Lactobacillus* spp., have been shown to secrete lactic acid and bacteriocins, contributing to microbiota balance and pathogen suppression. In vitro studies have shown that *Lacticaseibacillus rhamnosus* GG effectively inhibits the growth and adherence of several pathogenic bacteria, including *Salmonella*, *Shigella*, *Escherichia coli*, and *Streptococcus* species, highlighting its potential as a broad-spectrum probiotic with antimicrobial properties [218]. Some probiotic strains, such as *Ligilactobacillus salivarius* NRRL B-30514, isolated from chicken ceca, have demonstrated potential for producing bacteriocins that inhibit pathogenic bacteria. Specifically, studies have shown that this strain significantly reduces the presence of *Campylobacter jejuni* in the intestinal environment [219]. Although poorly documented, there is growing evidence suggesting that LAB, particularly those capable of producing bacteriocins or adhering to host cells, may have the potential to modify the microbiota of the teat apex and reduce the proliferation of pathogenic bacteria. Isolates from the genera *Lactobacillus* and *Lactococcus* have been evaluated for their ability to colonize the epithelium of the teat apex, where they demonstrated inhibitory activities against pathogenic bacteria such as *Staphylococcus aureus*, *Streptococcus uberis*, and *Escherichia coli* [220]. Bacteriocin-mediated effects of probiotic strains on pathogen inhibition have been demonstrated, as seen in Figure 3. Moreover, examples of animal derived bacterial strains and bacteriocins they produce are listed in Table 5.

#### 3.2.2. Organic Acids, Vitamins, and Exopolysaccharides

Animal-derived probiotic bacteria can produce organic acids such as lactic acid, acetic acid, and butyric acid, which acidify the gut environment and inhibit the growth of pathogenic bacteria. The production of amino acids by gut bacteria plays a critical role in the synthesis of SCFAs and the regulation of host metabolism. Bacteria in the gut produce several amino acids de novo, which act as precursors for SCFAs, substances that assist in the fermentation of undigested carbohydrates and influence the host’s physiology. These amino acids and their derived metabolites, including SCFAs, are known to regulate the metabolism of carbohydrates and lipids, ultimately contributing to the overall health of the host [225]. Additionally, LAB are involved in the proteolysis of casein molecules, producing small peptides and amino acids, which further contribute to various metabolic processes [226]. These acids also serve as a source of energy for the host and can modulate the immune response. The *Bifidobacterium* species and *Lactobacillus* species (*Lacticaseibacillus rhamnosus* GG, *Lacticaseibacillus rhamnosus* HA-114, among other animal-derived strains) produce lactic acid, which lowers the pH of the gut, creating an inhospitable environment for many pathogens [227].

Numerous different bacteria produce essential vitamins that are incredibly beneficial for the host’s organism. A summary of the bacterial strains and the vitamins they produce is to be found in Table 6.

Exopolysaccharides (EPSs) produced by probiotic bacteria, particularly LAB, have garnered attention due to their various health benefits, including immunostimulation, antitumor effects, antioxidant activity, and cholesterol-lowering properties. EPSs are synthesized through the action of enzymes such as glycosyltransferases and glycantransferases, which convert sugar nucleotide precursors into polysaccharides [237]. Among LAB, *Lactobacillus helveticus* has shown antitumor effects against cancer cell lines, including HepG-2, BGC-823, and HT-29 [238].

## 4. Factors Influencing the Safety and Effectiveness of the New Strains

Before implementing new strains of probiotics into both human and veterinary medicine, it is of great importance to thoroughly test them and understand the possible risks associated with their use. Unfortunately, the fact that products based on probiotic bacterial strains have been tested does not rule out the occurrence of adverse reactions following their administration. Such a situation sometimes occurs after the use of preparations containing *Clostridium* spp. strains. In Japan, a case was reported of an elderly man dying of bacteremia, which developed after consuming a commonly used (and one of the 10 prescribed) probiotics containing strains from the species *Clostridium butyricum* [239]. New, untested strains could pose a similar threat to the health of future consumers. In order to prevent this, all new strains must go through a rigorous series of testing. There were also particular cases where the administration of *Lactobacillus* probiotics has been associated with developing infections in immunocompromised patients [240]. In addition to what is written above, probiotic therapy may pose a threat when administered to patients with CKD. Tryptophan metabolites produced by gut microbiota, such as indoxyl sulfate, have been implicated in the progression of chronic kidney disease due to their pro-inflammatory and oxidative properties [241]. The role of probiotic therapy in modulating these metabolites remains contentious. Some studies caution against probiotic use, suggesting it may elevate levels of tryptophan-derived uremic toxins, potentially accelerating CKD progression [242]. Conversely, other research indicates that specific probiotic formulations can reduce these toxins, thereby exerting a protective effect on renal function [243].

A study from D’Agostin (2021) [244] identified 49 cases of probiotic-associated invasive infection in children, with sepsis being the most frequently reported outcome. Importantly, the majority of these cases occurred in infants under 2 years of age who had underlying risk factors, such as prematurity or the presence of indwelling intravenous catheters. Fortunately, 94% of affected children responded successfully to antimicrobial therapy, suggesting that early detection and treatment are effective.

The essential components required to establish the safety of a probiotic are the correct identification of the bacteria from the samples obtained using modern techniques, followed by the determination of the ability of these bacteria to colonize the relevant niche of the organism, the characterization of their resistance and sensitivity to various agents, and their stability and possible pathogenicity.

### 4.1. Stability and Safety Testing of New Probiotic Strains

The stability of probiotic strains is an area in which not much research has been conducted. The high stability of the genome is a factor that ensures that passage through multiple hosts and long-term colonization keep the strains in their original, unchanged form. Sequencing the whole genome is required to obtain all needed information, and such studies have not been conducted in multiple strains, leaving this area open for further research [245].

In order to register new strains of probiotics, they must go through a series of tests to prove that they are safe for human and animal consumption. Tests can be divided into three groups: in vitro safety assessments, in vivo animal studies, and human clinical trials.

To check for carrying antibiotic resistance genes, the most common method is Minimum Inhibitory Concentration (MIC) testing. Hemolysis tests, gelatinase and protease activity, biofilm formation assay, adhesion and invasion assays, toxin gene screening, and DNAse tests enable potential virulence factor identification. Metabolic activity screening ensures no production of harmful compounds like D-lactate. In vitro test results may differ from those received after an in vivo method of testing [246].

If the in vitro tests are promising, animal models (usually rodents) are used to assess safety. These studies provide insight into the effects of probiotics on a living organism and its microbiota, offering data that can not be obtained during in vitro testing. Rodents such as mice and rats are mainly chosen for this purpose due to their well-known anatomy and physiology. Gnotobiotic animals are used for testing the effect of probiotics on chosen microbes by intentionally inserting them into the animal organisms that were previously free of germs. To test the impact of probiotics on vulnerable populations, immunocompromised models are used [247].

To identify any toxic response, both single and repeated doses should be given to the model. This will result in checking the risk of both methods of supplementation, which can inhibit acute, sub-chronic, and chronic reactions to the given strain, identifying any kind of toxic response. Bacterial translocation has to be checked in order to observe the probiotics’ possible route into the body. Potential pathogenicity can be indicated by the translocation of the strain to sterile tissues like the liver, spleen, or the bloodstream. Infectivity in vulnerable populations is checked using immunocompromised models [248]. Both ante-mortem and postmortem examination of the samples is crucial. Histopathological and microbiological examinations of the sample are conducted to detect any adverse effects or bacterial translocation [249].

Conducting human clinical trials is a pivotal step in validating the safety of new probiotic strains. Randomized Controlled Trials (RCTs) are the gold standard for clinical research. The length of the study should enable the observation of effects on the human body. Immunocompromised individuals require additional testing. The results have to be thoroughly analyzed, and a report of the study should be written to finish the trial. Any adverse effects should be reported and analyzed closely [250].

To ensure the quality, safety, and efficiency of new probiotic strains, test method guidelines need to be met. Several organizations have developed rules for the tests. The Food and Agriculture Organization (FAO) and the World Health Organization (WHO) prepared the FAO/WHO Guidelines for the Evaluation of Probiotics in Food, which provides information on the testing methods for probiotics intended for food use. The International Probiotics Association (IPA) wrote guidelines focusing on the production and marketing of probiotic-containing products. In Europe, the guidelines of the European Federation of Associations of Health Product Manufacturers (EHPM) must be met [251].

Conducting in vivo studies on both animal models and humans requires adherence to strict ethical standards. Animal welfare, as well as scientific validity, are key factors in obtaining data.

### 4.2. Effectiveness

The implementation of new probiotic strains requires a complicated series of tests, including those examining the effectiveness of potential new strains. In order to determine the effects of the strain and register them for human or animal consumption, multiple factors need to be checked, including clinical evidence of probiotic therapy, the mechanism of action, and interactions [252]. Without proving the effectiveness of new strains, implementing them for consumption is redundant [253].

The mechanism of action (MoA) is crucial in probiotic research and implementation. Without determining the MoA, probiotics may be inconsistently effective, unsafe, or not effective at all. Different strains have different MoAs, which influence their effects. Subtle differences between species may cause crucial differences in their functioning. Because of this, seemingly similar strains can act differently. Identifying the MoA ensures the selection of strains based on their specific medical outcomes, making targeted therapy possible. Establishing the MoA is one of the steps required by national federations responsible for probiotic analysis, which has to be conducted before registering them. In a study by Mingkang et al., the mechanisms of action of probiotics are divided into three main groups: the production of bioactive compounds, the regulation of the gastrointestinal microbiome, and the modulation of the immune and nervous systems [254].

The effectiveness of probiotics largely depends on strain specification, dose, and the formula of application. Proper dosing and formulation ensure survival, colonization, and therapeutic benefits, reducing the risk of adverse effects or failing to deliver desired results [255]. The recommended daily dose generally ranges from 10^6^ to 10^12^ CFU. However, it can vary widely based on the specific strain of probiotics, the health problem being treated, and the patient’s health status and general condition. Probiotics with lower CFUs (1–5 billion CFUs per day) are recommended for maintaining the general proper condition of gut microbial flora or as the starting point for probiotic therapy. On the contrary, for conditions like irritable bowel syndrome (IBS), IBD, or antibiotic-associated diarrhea, higher doses (10–50 billion CFUs per day) are proven to be more effective [256]. For example, research on the probiotic *Lacticaseibacillus rhamnosus* Lcr35 revealed that its effect on human dendritic cells is dose-dependent. At higher doses, the probiotic-induced semi-maturation of dendritic cells and a strong pro-inflammatory response are characterized by increased production of pro-Th1/Th17 cytokines. This finding underscores the necessity of precise dosing to achieve the desired immunomodulatory effects [257].

## 5. Conclusions

Probiotics derived from animal origin present a promising tool in both human and veterinary medicine, offering a wide array of health benefits across species. From mitigating gastrointestinal disorders, enhancing immune responses, and improving wound healing to enhancing livestock productivity, their applications are extensive and continue to evolve. However, despite their therapeutic potential, probiotics remain underappreciated, not only for their benefits but also for the risks they may pose if not thoroughly investigated and carefully applied.

While numerous strains are already in clinical or commercial use, ongoing research continues to unveil new candidates with unique properties, including enhanced resilience to environmental conditions with greater precision and efficacy. These discoveries pave the way for tailored probiotic therapies that could target specific health conditions with greater precision and efficacy. Yet, such advancements need rigorous safety evaluations, including in vivo and in vitro studies, to assess factors like pathogenicity, drug resistance, and stability.

Looking ahead, the future of probiotic research lies in striking a careful balance between innovation and safety. Probiotics should not be viewed as universally benign; individual patient history, potential interactions with medications, and strain-specific characteristics must all be considered. The benefit/risk ratio must guide clinical decisions, particularly as many probiotic products remain available over the counter without prescription. This widespread accessibility, while advantageous, also calls for increased public awareness and potentially stricter regulatory guidelines to ensure that probiotic use aligns with scientific evidence and patient safety.

To become more beneficial for the future, the ongoing research on probiotics should focus on the development of standardized protocols for probiotic strain identification, characterization, and clinical testing to ensure reproducibility and comparability across studies. Investigating host-specific responses, including differences in gut microbiota composition, immune modulation, and pharmacokinetics, can help tailor probiotic therapies to individual patients or animal species. Expanding longitudinal studies and real-world trials will further clarify long-term safety, efficacy, and optimal usage parameters, guiding both regulatory policy and clinical practice.

Ultimately, novel animal-origin probiotics represent a frontier of opportunity within the One Health framework, linking human and animal well-being. Their future development holds immense promise, but their responsible integration into healthcare requires continued research, thoughtful risk assessment, and a commitment to prioritizing patient safety alongside therapeutic innovation.

## Figures and Tables

**Table 1:** Effects of application of some probiotic strains used in cats and dogs, along with their origin.

Probiotic Strain	Effect on Host Organism	Origin of Isolation
*Bifidobacterium animalis* AHC7 (2 × 10^10^ CFU/day)	In young adult dogs with acute diarrhea, supplementation with *B. animalis* AHC7 resulted in a significant reduction in diarrhea compared to the placebo group	Canine source [96]
*Bifidobacterium animalis* B/12 (1 mL of 1.04 × 10^9^ CFU/mL)	When administered to healthy dogs, decreased serum triglyceride and albumin concentrations while increasing ALT and ALP levels. It also resulted in an increase in acetic, acetoacetic, and valeric acids in the feces	Canine source [97]
*Enterococcus faecium* DSM 32820 (10^9^ CFU/day)	In healthy dogs, supplementation resulted in a decrease in serum glucose concentration, indicating a potential role in metabolic health	Canine source [98]
*Enterococcus faecium* SF68 (5 × 10^9^ CFU/day):	In kittens, supplementation resulted in a significant increase in the percentage of CD4+ lymphocytes, suggesting a role in immune function enhancement	Feline source [99]
*Enterococcus hirae* (2.85–4.28 × 10^8^ CFU/day)	In kittens, it promoted intestinal colonization and fecal shedding of live *E. hirae*. This strain ameliorated the effects of atypical Enteropathogenic *E. coli* (EPEC) infection, improving intestinal function and reducing water loss	Feline source [100]
*Enterococcus faecium* SF68 (5 × 10^8^ CFU/day)	In dogs with diarrhea, the exact probiotic strain combined with metronidazole improved diarrhea and eliminated *Giardia* cysts more effectively than metronidazole alone	Feline source [99]
*Lactobacillus acidophilus* D2/CSL (CECT 4529) (5 × 10^9^ CFU/kg of food)	In healthy adult cats, supplementation improved fecal quality and increased *Lactobacillus* species while reducing coliform bacteria counts.	Conventional foods such as milk, yogurt, and dietary supplements [101]
*Lactobacillus casei* Zhang, *Lactobacillus plantarum* * P-8, and *Bifidobacterium animalis* subsp. *Lactis* V9 (2 × 10^9^ CFU/g)	In dogs across various age groups, these probiotics significantly promoted feed intake and weight gain. They enhanced serum IgG levels, increased fecal sIgA, and reduced TNF-α levels. They also contributed to a better balance of gut bacteria	*Lactobacillus casei* Zhang (koumiss) *Lactobacillus plantarum* * P-8 (fermented dairy products in China); *Bifidobacterium animalis* subsp. Lactis V9 (feces of a healthy Mongolian child) [102]
*Lactobacillus fermentum* AD1 (3 mL of 10^9^ CFU/mL)	In healthy dogs, supplementation significantly increased blood lipid and protein levels, lowered blood glucose, and increased the abundance of lactobacilli and enterococci in feces	Canine source [103]
*Lactobacillus fermentum* CCM 7421 (10^7^–10^9^ CFU/day)	In dogs with gastrointestinal disorders, improved blood parameters, including total protein, cholesterol, and ALT levels. This strain also increased lactic acid bacteria populations and reduced clostridia levels while normalizing fecal consistency	Canine source [98]
*Lactobacillus johnsonii* CPN23 (2.3 × 10^8^ CFU/day)	In adult female Labrador dogs, supplementation enhanced nutrient digestibility, increased SCFA concentrations, and reduced fecal ammonia levels, indicating benefits for gastrointestinal health	Canine source [104]
*Lactobacillus johnsonii* CPN23 (10^8^ CFU/mL, 0.1 mL/kg BW)	In adult female dogs, supplementation decreased plasma glucose and cholesterol levels and improved the HDL/LDL ratio	Canine source [105]
*Lactobacillus murinus* LbP2 (5 × 10^9^ CFU/day)	In dogs suffering from canine distemper virus (CDV)-associated diarrhea, supplementation led to improvements in fecal consistency, mental status, and appetite	Canine source [106]
Lactobacillus plantarum * (1 × 10^8^ CFU/mL)	Administered through mare’s milk; improved symptoms of chronic gingivostomatitis in cats, reducing inflammation and oral pain	Mare’s milk [107]
Proviable^®^-DC (7 bacterial species)	This multistrain probiotic product, containing seven bacterial species, improved stool consistency and alleviated diarrhea symptoms in both cats and dogs. It also increased the abundance of probiotic bacteria in the feces of healthy cats	Multistrain probiotic product [108]

* Currently used species name: *Lactiplantibacillus plantarum*.

**Table 2:** Effects of the application of selected probiotic strains used in pigs, along with their origin.

Probiotic Strain and Dosage	Host’s Specie and Age	Effect on Host Organism	Origin of the Probiotic Strain
*Bifidobacterium animalis* subsp. *lactis* JYBR-190, 1 × 10^9^ CFU/kg of feed	Piglets, 21 days old	Improved intestinal development, enhanced antioxidant activity, modulated gut microbiota (increase in beneficial bacteria; decrease in pathogens), and reduced incidence of diarrhea in weaned piglets	Swine gastrointestinal tract [152]
*Bacillus subtilis* PB6, 4 × 10^8^ CFU/kg of feed	Sows and piglets, precise age not specified	Increased the litter sizes, litter weights, lactation survival rate, and litter weight gains at weaning	Intestines of healthy chickens [153]
*P. acidilactici* FT28, 200 g fermented feed/pig/day	Female piglets, 28 days old	Increased feed intake, decreased serum concentration of glucose, decreased serum concen- tration of triglycerides and cholesterol,	Weaned pig- let feces [154]
Bokashi ^®^ (*S. cerevisiae*, *L. casei*, *L. plantarum*, *E. faecium*, *E. faecalis*, *Bifidobacterium bifidum*, *Bifidobacterium* *pseudolongum*, *B.* *licheniformis*, *B. cereus* *var toyoi*, *B. subtilis*, *C.* *butyricum*), dosage not specified	Sows, precise age not specified	Significantly higher concentration of IL-2, IL-4, IL-6, and IL-10 * in colostrum; increased litter size, lactation length; higher birth weight of newly born piglets	Multistrain preparation [155]

* IL—interleukin.

**Table 3:** Effects of the application of selected probiotic strains used in poultry, along with their origin.

Probiotic Strain and Dosage	Host’s Specie and Age	Effect on Host Organism	Origin
*Enterococcus faecium* PNC01, 1 × 10^9^ CFU/kg feed	Broiler chickens (1 to 42 days old)	Increased villus height and crypt depth, altered cecal microbiota (increased *Firmicutes* and *Lactobacillus*; decreased *Bacteroides*), inhibited *Salmonella typhimurium* invasion of intestinal epithelial cells	Intestinal mucosa of broiler chickens [160]
*Enterococcus faecium* AL41, 1 × 10^9^ CFU/day per bird	7-day-old broilers	Produced bacteriocin (Enterocin M); administration resulted in higher percentage of phagocytic activity in the gastrointestinal tract	Poultry gut isolate [161]
*Bacillus subtilis* PB6, 1 × 10^8^ CFU/kg feed	Broiler chickens (1 to 42 days old)	Increase in body weight and daily weight gain, improved villus height and crypt depth	Chicken’s gastrointestinal tract [158]
*Bacillus subtilis* DSM29784, 1 × 10^9^ CFU/kg feed	Broiler chickens, precise age not specified	Low levels of lesion scores (in correlation to necrotic enteritis), improved villus height	Avian gastrointestinal tract [162]

**Table 4:** Effects of the application of selected probiotic strains used in cattle, along with their origin.

Probiotic Preparation and Dosage	Host’s Specie and Age	Effect on Host Organism	Origin
*Lactobacillus casei* Zhang and *Lactobacillus plantarum* P-8, 10^9^ CFU/day	Lactating Holstein cows, ~3–5 years old	Increased milk yield while reduced somatic cell count by positively affecting the composition of the rumen microbiota	Human gastrointestinal tract and bovine gastrointestinal tract [175]
*Enterococcus faecium* M74, 1 × 10^9^ CFU/day	Piglets, ~3–8 weeks old	Positive effect with significant improvements in body weight and daily weight gain over the entire study period of probiotic treatment (62 days); reduced incidence of diarrhea	Swine intestines [176]
*Enterococcus faecium* EGY_NRC1, 2 × 10^9^ CFU/head	Lactating Holstein cows, ~4–6 years old	Improved digestibility of dry matter, neutral detergent fiber (NDF), and acid detergent fiber; increased glucose levels and reduced cholesterol	Bovine milk and fermented milky products [177]
*Lactobacillus gallinarum* JCM 2011(T), *Streptococcus infantarius* subsp. *coli* HDP90246 (T), *Streptococcus salivarius* subsp. *thermophilus* ATCC 19258(T), *Streptococcus equinus* ATCC 9812(T), *Saccharomyces cerevisiae*_1, 5 × 10^8^ CFU/kg	Cattle, ~6–12 months old	Increased body weight and daily weight gain, increase in hemoglobin, packed cell volume (PCV), red blood cells count, and mean corpuscular volume (MCV)	Bovine milk and milk products [178]
*Megasphaera* *elsdenii* SA3, 1 × 10^9^ CFU/cow/day	Lactating cows, ~3–5 years old	Decreased plasma lactate dehydrogenase	Bovine rumen [179]

**Table 5:** Examples of animal-derived bacterial strains and the bacteriocins they produce.

Bacterial Strain	Animal Source	Bacteriocins Produced	Target Pathogens
*Ligilactobacillus salivarius* P1CEA3, PG21	Swine (gastrointestinal tract)	P1CEA3: Salivaricin B, Abp118α, Nisin S (class I), Abp118β; PG21: Bactofencin A, Salivaricin Tα LP, Salivaricin Tβ LP, Gassericin T/LactacinF lafA LP, Plantaricin NC8α LP, Plantaricin NC8β LP, Plantaricin Sα LP, Plantaricin Sβ LP	*Staphylococcus aureus*, *S. suis* [221]
*Enterococcus faecium* SH528, SH632	Chicken (gut)	Enterocin A, B, L50, P	*Listeria monocytogenes*, *Clostridium perfringens* [222]
*Pediococcus pentosaceus* SH740	Chicken (gut)	Pediocin PA-1	*Listeria monocytogenes*, *C. perfringens* [222]
*Escherichia coli* (72 various strains)	Fecal samples obtained from various livestock (cows, pigs, rabbits, poultry)	Colicins and microcins (including mccV, mccL, Ia, Ib, E1, B, K, A, Y, N, U, S4, mccB17, mccC7)	*E. coli*, *Salmonella enterica* [223]
*E. faecium* HC121.4, HC161.1, *E. mundtii* HC26.1, HC56.3, HC73.1, HC73.2, HC112.1, HC121.4, HC147.1, HC155.2, HC161.1, HC165.3, HC166.3, HC166.5, and HC166.8	Sheep and goat colostrum	Mundticin, enterocins	*L. monocytogenes*, *S. aureus*, *E. coli*, *P. aeruginosa*, *S. typhimurium*, *B. cereus*, as well as LAB [224]

**Table 6:** A summary of the bacterial strains and the vitamins they produce.

Vitamin	Function	Bacterial Origin
Thiamine (Vitamin B1)	Needed for nucleic acid, fatty acid, and aromatic amino acid synthesis	Produced by *Bifidobacterium* species [228]
Pyridoxine (Vitamin B6)	Crucial for early nervous system development	Produced by *Bifidobacterium* species [229]
Folic acid (Vitamin B9)	Essential for nucleic acid synthesis, amino acid conversions, and antioxidant functions	Produced in large quantities by gut microbiota; however, not all probiotic strains can synthesize folate, as *Lactiplantibacillus plantarum* * lacks this ability [230]
Vitamin B12	Important for blood formation and nervous system function	Primarily produced by bacteria such as *Lactobacillus reuteri* [231] and *Propionibacterium shermani* [232]
Menaquinone (Vitamin K2)	Crucial for blood clotting	Produced by intestinal bacteria [233]: *E. coli*, *Bacteroides* species, and some Gram-positive, anaerobic, non-spore-forming bacilli [234]
Vitamin A	Supports vision, immune function, cell growth, and skin health [235]	*Escherichia coli* Nissle 1917 (EcN) has been genetically engineered to produce β-carotene, increasing vitamin A levels in the intestine [236]

* previously known as *Lactobacillus plantarum*.

## Data Availability

No new data were created or analyzed in this study. Data sharing is not applicable to this article.

## Abbreviations

The following abbreviations are used in this manuscript:

ACE	Angiotensin-converting enzyme
ACD	Allergic contact dermatitis
AD	Alzheimer’s disease
AFLP	Amplified Fragment Length Polymorphism
ARGs	Antibiotic resistance genes
ASD	Autism spectrum disorder
ATP	Adenosine Triphosphate
BSH	Bile salt hydrolase
BL999	*Bifidobacterium longum* (strain 999)
CLA	Conjugated linoleic acid
CDI	*Clostridioides difficile* Infection
CFU	Colony-forming unit
CKD	Chronic kidney disease
CNS	Central nervous system
COX-2	Cyclooxygenase-2
CPEK	Canine progenitor epidermal keratinocytes
DNA	Deoxyribonucleic Acid
EcN	*Escherichia coli* Nissle 1917
EHEC	Enterohemorrhagic *Escherichia coli*
EHPM	European Federation of Associations of Health Product Manufacturers
ENS	Enteric nervous system
EPSs	Exopolysaccharides
EPEC	Enteropathogenic *Escherichia coli*
FAO	Food and Agriculture Organization
FHV-1	Feline herpesvirus type 1
FIC	Feline Idiopathic Cystitis
FISH	Fluorescent In Situ Hybridization
GALT	Gut-associated lymphoid tissue
GBA	Gut–brain axis
GIT	Gastrointestinal tract
GFR	Glomerular filtration rate
GLUT2	Glucose transporter 2
H. pylori	*Helicobacter pylori*
HPA	Hypothalamic-pituitary-adrenal
HIF	Hypoxia-inducible factor
IBD	Inflammatory bowel disease
IBS	Irritable bowel syndrome
Is	Indoxyl sulfate
IgA	Immunoglobulin A
LAB	Lactic acid bacteria
LG2055	*Lactobacillus gasseri* SBT2055
LcS	*Lactobacillus casei* strain Shirota
MIC	Minimum Inhibitory Concentration
MDR	Multidrug-resistant
MRSA	Methicillin-resistant *Staphylococcus aureus*
MOA	Mechanism of action
UTI	Urinary tract infection
pCS	p-Cresyl Sulfate
RCTs	Randomized Controlled Trials
ROS	Reactive oxygen species
rUTIs	Recurrence of Urinary Tract Infections
SCFA	Short-chain fatty acids
sIgA	Secretory Immunoglobulin A
STT	Standard triple therapy
TC	Total cholesterol
T2DM	Type 2 diabetes mellitus
TGF-β1	Transforming Growth Factor Beta 1
Tregs	T regulatory cells
UPEC	Uropathogenic *Escherichia coli*
VRE	Vancomycin-resistant Enterococci
VBNC	Viable but Non-Culturable
VVC	Vulvovaginal Candidiasis
WHO	World Health Organization
